# Bovine colostrum and its potential contributions for treatment and prevention of COVID-19

**DOI:** 10.3389/fimmu.2023.1214514

**Published:** 2023-10-16

**Authors:** Hatice Duman, Sercan Karav

**Affiliations:** Department of Molecular Biology and Genetics, Canakkale Onsekiz Mart University, Canakkale, Türkiye

**Keywords:** bovine colostrum, bioactive compound, immunomodulation, SARS-CoV-2, COVID-19, antiviral activity, health benefits

## Abstract

Bovine colostrum (BC) is the initial milk an animal produces after giving birth, particularly in the first few days. Numerous bioactive substances found in BC, including proteins, enzymes, growth factors, immunoglobulins, etc., are beneficial to human health. BC has a significant role to play as part of a healthy diet, with well-documented health and nutritional advantages for people. Therefore, the use of BC and its crucial derivatives in the development of functional food and pharmaceuticals for the prevention of several diseases such as gastrointestinal and respiratory system disorders is becoming increasingly popular around the world. A novel coronavirus severe acute respiratory syndrome coronavirus 2 (SARS-CoV-2) was identified as the cause of a cluster of pneumonia cases that is called Coronavirus Disease 2019 (COVID-19) in China. After the first SARS-CoV-2 virus-related fatality was announced, the illness quickly spread throughout China and to other continents, causing a pandemic. Since then, numerous studies have been initiated to develop safe and efficient treatments. To prevent viral infection and potential lingering effects, it is important to investigate alternative treatments for COVID-19. Due to its effective bioactive profile and its immunomodulatory roles in biological processes, BC might be considered a promising approach to assist in combating people affected by the SARS-CoV-2 or prevention from the virus. BC has immunomodulatory effects because to its high concentration of bioactive components such as immunoglobulins, lactoferrin, cytokines, and growth factors, etc., which might help control immunological responses, potentially fostering a balanced immune response. Furthermore, its bioactive components have a potential cross-reactivity against SARS-CoV-2, aiding in virus neutralization and its comprehensive food profile also supplies important vitamins, minerals, and amino acids, fostering a healthy immune system. Hence, the possible contributions of BC to the management of COVID-19 were reviewed in this article based on the most recent research on the subject. Additionally, the key BC components that influence immune system modulation were evaluated. These components may serve as potential mediators or therapeutic advantages in COVID-19.

## 1 Introduction

Bovine colostrum (BC) is the initial secreted milk from bovine mammary glands during the first few days after calving (1). It contains several bioactive nutrients and immunological compounds that are necessary for the newborn’s nourishment as well as growth and development (2). Colostrum is incredibly rich in bioactive substances such immunoglobulins (IgG, IgA, IgE, IgD, IgM), growth factors, hormones, lysozymes, lactoferrin (Lf), and lactoperoxidase (LPO). Additionally, it has higher levels of lipids, proteins, minerals (phosphates, citrates, etc.), and vitamins (water and fat-soluble) (**Table 1**) (1, 15, 16). With the exception of lactose content in BC, the concentration of these components is highest in the first few days after postpartum, thereafter rapidly decreasing over the next three days (17).

**Table 1:** Concentration of bovine colostrum main components.

	Concentration (g/L)			
Components	Bovine Milk	Bovine Colostrum	Biological Activity	References
Protein	33	250		(3)
Caseins	28	26	Effective in inflammation, immunomodulator, ion carrier, precursor of bioactive peptides	(3, 4)
Whey	5	30-200		(3)
β-Lactoglobulin	3.3	8.0	Retinol carrier, binding fatty acids, antioxidant, precursor for bioactive peptides	(3, 5)
α-Lactalbumin	1.2	3	Lactose synthesis in mammary gland, Ca carrier, immunomodulator, anticarcinogenic, precursor for bioactive peptides, supports cognitive abilities	(3, 5)
Immunoglobulin	0.5-1	20-150	Immune protection, antimicrobial, precursor for bioactive peptides	(3, 6)
*IgG_1_*	0.58	46.40		(7)
*IgG_2_*	0.06	2.87		(7)
*IgA*	0.08	5.86		(7)
*IgM*	0.09	6.77		(7)
Lactoferrin	0.1	1.5	Antimicrobial, antioxidative, anticarcinogenic, effective in inflammation, immunomodulator, iron transport, regulation of cell growth, precursor for bioactive peptides	(3, 8)
Growth factors	50 µg-40 mg/mL	< 1 µg – 2 mg/mL	Regulation of cell growth, survival, differentiation, and protection and repair of intestinal cells, immunomodulator	(3, 9)
*IGF-I (µg/L)*	0.049-2	<0.002-0101		(10)
*IGF-2 (µg/L)*	0.15-0.6	0.002-0.1		(10)
*TGF- β1 (µg/L)*	0.0124-0.0426	0.0008-0.0035		(10)
*TGF- β2 (µg/L)*	0.15-1.15	0.013-0.07		(10)
Lysozyme	0.0004	0.0004	Antimicrobial, neuroprotective, synergistic effect with Igs and Lf	(11, 12)
Lactoperoxidase	0.03	0.02	Antimicrobial, synergistic effect with Igs and Lf	(11, 12)
Lactose	46	30-40		(3)
Oligosaccharides	0.1-0.2	0.7-1.2	Support immune and intestinal system, antimicrobial, prebiotic activity, promote brain development	(3, 13)
Lipids	35-42	40-60	Immunomodulator, antimicrobial, effective in inflammation	(3)
Minerals	7	7-10	Regulation of metabolism, support growth and development, maintain immune system	(3)
Vitamins			Regulation of metabolism, support growth and development, maintain immune system	
*Riboflavin (B2) (µg/mL)*	1.5–1.7	4.55–4.83		(14)
*Cobalamin (B12) (µg/mL)*	0.004–0.006	0.05–0.60		(14)
*Vitamin A (µg/100 mL)*	34	25		(14)
*Vitamin D (IU/g fat)*	0.41	0.89–1.81		(14)
*Tocopherol (E) (µg/g)*	0.06	2.92–5.63		(14)

Both internal and external factors have a considerable impact on the composition and quality of BC (15, 18). Major contributing factors including environment, individuality, season, humidity, breed, dry period, pre-partum nutrition, calving, etc., changes the BC composition and quality. Diseases such as mastitis are other factors that change the BC content and quality (19–24).

**Figure 1:**
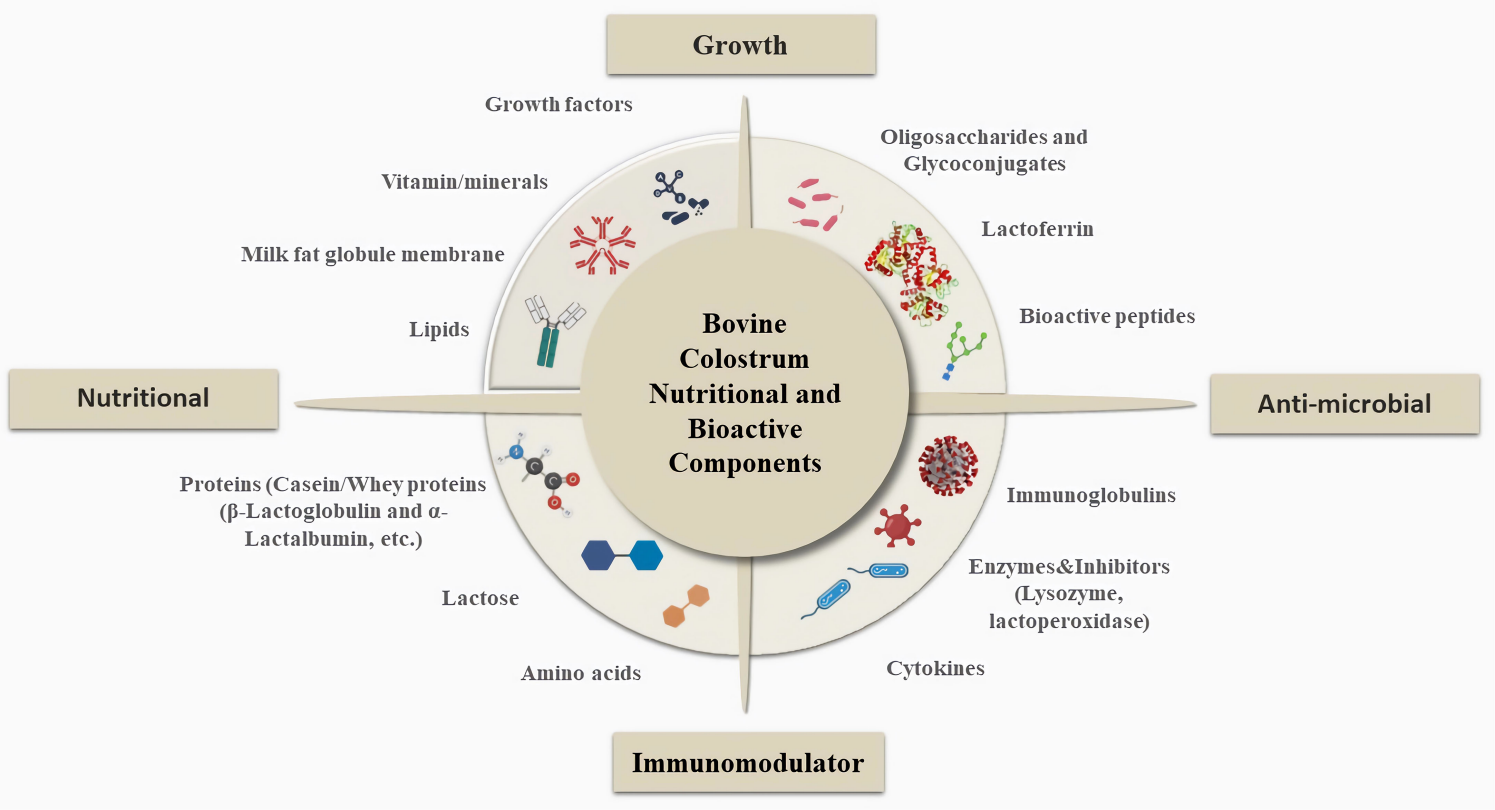
General Overview of Bovine Colostrum Composition and Important Roles. BC contains a variety of nutritionally and physiologically significant compounds and each active substance serves a specific purpose. Considering the general content of BC, the main nourishing part, such as lipids, vitamins-minerals, growth factors, proteins, and lactose, provides energy to newborn calves and supports calf growth, while the biologically active components, such as Igs, Lf, growth factors, etc., support the immune system and provide passive immunity to protect against several infections from pathogens, viruses, etc. In addition to these components, oligosaccharides and glycoconjugates, bioactive proteins, and peptides support calf development and also have antimicrobial properties (25, 26).

Nutritionally and biologically important substances are found in BC at different concentrations and each active substance has a distinct role based on their biological forms as well as types of action shown in **Figure 1**. Considering the general content of BC, the main nourishing part allows providing energy to the newborn calves, while the biologically active components such as immunoglobulins (Igs), Lf, growth factors, etc., support the immune system and provide passive immunity to protect from several infections from pathogens, viruses, etc. (**Table 1**) (15, 27).

A novel coronavirus “severe acute respiratory syndrome coronavirus 2 (SARS-CoV-2)” was identified as the cause of a cluster of pneumonia cases that is called “Coronavirus Disease 2019 (COVID-19) in Wuhan City, China, in late 2019. Within a few months, the disease spread across China and to various continents, causing a global pandemic (28). Since then, several research has been started to perform to find effective and safe treatments. Clinically, manifestations of COVID-19 show a wide heterogeneity, ranging from asymptomatic cases to mild symptoms, including fever, dry cough, headache, myalgia, dyspnea, and, in some cases, diarrhea, nausea, and vomiting. The gradual progression of this disease may lead to difficulty breathing, respiratory distress, pneumonia, cardiac, and neurological damage, and in severe cases, even multiple organ failure and death. It’s worth noting that the severity and duration of COVID-19 repercussions can vary greatly amongst people. While many people recover with no long-term complications, others may face serious health concerns. Additionally, the virus’s influence goes beyond health, influencing society in the form of economic downturns, disruptions in education, mental health issues, and changes in social behavior as a result of public health initiatives (29, 30). The treatment and prevention of the SARS-CoV-2 illness are still being researched, even though there are currently a number of vaccinations available. Additionally, due to the constant evolution of the virus, new viral strains may not be effectively recognized by vaccine-induced protection (31).

To sum up, prior to the development of the vaccine, it is urgently necessary to investigate alternative treatments for COVID-19 clinically advanced conditions without causing side effects in order to decrease viral infection, replication, and spread as well as mortality and to mitigate potential future outbreaks. Due to its effective bioactive profile and its immunomodulatory roles in biological processes, BC can be considered as a possible strategy to assist in treating people affected by SARS-CoV-2 (32).

Based on the most recent research on the topic, the potential contributions of BC to the clinical care of COVID-19 were discussed in this article. Additionally, the primary colostrum components that affect immune system modulation and may function as mediators of possible therapeutic benefits in COVID-19 disease were reviewed.

## 2 The main immunomodulating and bioactive components of bovine colostrum

### 2.1 Caseins

The biological characteristics of milk proteins have drawn more attention from scientists and industries such as the food and pharmaceuticals industry (33, 34). Colostrum has a significantly higher protein content than mature milk, mostly because of the higher concentrations of Igs and casein. Whey proteins and caseins, which are two types of milk proteins, can be distinguished based on their solubility in milk. A total of 150 g/L of protein are present in BC, of which 124 g/L are whey protein and 26 g/L are casein In comparison to casein content of milk, colostrum has a higher casein concentration and gets lower with each postpartum milking (34).

Caseins are proteins that control inflammation, and the immune system as well as they have antibacterial activity. As mineral binders’ proteins, they enhance digestion by producing a clot in the stomach, which shortens the time it takes for nutrients to enter the bloodstream and improves digestion (35). The various bioactive peptides derived from casein protein and its sub-forms (α, β, and κ) impart significant physiological properties to them. For example, the therapeutic peptide “RYLGY” was identified by Dash and Jaganmohan (2022) from cold plasma-treated S1-CN of bovine milk. This peptide may interfere with the SARS-CoV-2 spike protein’s ability to bind to the angiotensin-converting enzyme 2 (ACE2) receptors on cell membranes, preventing the virus from entering cells. *In-silico* docking studies showed that the peptide and ACE2-receptor binding domain complex had increased binding affinity and electrostatic interactions (36).

On the other hand, glycomacropeptides (GMP) which is also known as caseinomacropeptide, is a type of C-terminal glycopeptides isolated from κ-casein. The biological functions of GMP have attracted more attention in recent decades (10). Numerous *in-vitro* studies have demonstrated that GMP inhibits the adhesion of cariogenic *Str. mutans* and *Str. sobrinus* bacteria as well as influenza virus and counteracts the microbial toxins of *Escherichia coli* (*E. coli*) and *Vibrio cholerae*. Furthermore, GMP modifies immunological responses, promotes the growth of beneficial microorganisms such as bifidobacteria, suppresses gastric hormone activities as well as controls blood flow through hypertension and antithrombotic activity (37, 38). The usage of GMP can be a possible strategy to improve the treatment against COVID-19. These results imply that casein and its derivatives may be particularly effective in combating COVID-19, but additional studies are required to properly understand the influence of casein and its peptides as effective targets for COVID-19 (3, 39).

### 2.2 Lactoferrin (Lf)

Lf is a highly glycosylated whey protein found in bovine milk, with considerably high quantities in BC (**Table 1**). It has the potential to bind to iron and interact with several pathogens, making it the first line of defense in the mucous membranes of the body (40, 41). It has antibacterial, antiviral, antifungal, antiparasitic, and anti-inflammatory properties, as well as being implicated in innate and adaptive immune modulation (42, 43). Lf has antiviral activity against DNA and RNA viruses (44, 45), and the exact mechanism of its activity against bacterial and viral infections has been highlighted for the prevention of diseases (46).

The underlying mechanism of the antiviral role of Lf in BC is the inhibition of viral entry by attaching to the cell surface and/or viral molecules (47). Up to date, various studies and clinical trials regarding the antiviral activity of Lf derived from milk against different viruses including SARS (48), type I and II herpes simplex virus (HSV) (49, 50), human immunodeficiency virus (HIV) (51), rotavirus, influenza virus, parainfluenza virus, cytomegalovirus, and enterovirus (52–55).

The potential role of BC for the clinical treatment of COVID-19 is based on its an-inflammatory, antibacterial, and antiviral properties as well as on its ability to boost the human innate and adaptive immune systems. Considering the literature, clinical studies suggested that bioactive components of BC, particularly Lf, have considerable antiviral activity, which could assist to delay the progression of COVID-19 (56, 57).

Numerous clinical trials are being conducted to examine the anti-SARS-CoV-2 activity of Lf *in-vivo* in light of the findings from *in-vitro* and *in-silico* research. Lf may be used alone or in conjunction with other medications to treat COVID-19 disease (32). Lf from BC was found to be effective against SARS-CoV-2 viruses, similar to its effectiveness against Zika and Chikungunya viruses (56). In an *in-vivo* preliminary study, the antiviral effect of liposomal bovine Lf (LLf) was investigated for three months. For this investigation, mild-to-moderate and asymptomatic COVID-19 patients were chosen, and they were classified three main groups including patients (hospitalized and home isolation) who had oral and intranasal LLf; a hospitalized patient who recruited with standard protocol; and remaining patients in home isolation without any medical treatments. Control groups were also specified for the exact analysis. According to study results, LLf-treated patients recovered as compared to other groups. It was concluded that there was a decrease in serum ferritin, interleukin 6 (IL-6), and D-dimer levels. Given the high Lf content of BC, the use of this bioactive component for the effective treatment of COVID-19 and related infections may be a promising approach (58).

In addition to the antiviral activity of Lf, the anti-inflammatory feature of Lf could be effective in moderating the cytokine storm activation seen in severe COVID-19, which causes several health problems such as pulmonary edema and failure, hepatic, cardiac, and renal damage (59). In prospective observational research by Serrano et al. (2020), the impact of LLf supplementation was tested in 75 COVID-19 patients over 10 days. Based on the study, LLf (32 mg of Lf/10 mL together with 12 mg of vitamin C) was given to the patient. 10 mg/mL of zinc solution was also administered twice or three times per day. On the other hand, 12 patients were given only LLf as a control group, while 256 people who had contact with COVID-19 patients were also administered half of the treated dosage. Within the first 4-5 days of treatment, all patients recovered completely and quickly. The reduction of incidence of disease symptoms such as dry cough, headache, fatigue, anosmia, and ageusia was observed. They concluded LLf carries the potential for the prevention and treatment of COVID-19 (44).

The impact of Lf treatment on immune response and infection of SARS-CoV-2 was also investigated *in-vitro* study. Researchers used real-time quantitative reverse transcription PCR (qRT-PCR) to examine the expression of antiviral immune responses in infected and uninfected Caco-2 intestinal cell lines that had received Lf. As a result of the assessment, the expression of IFNA1, IFNB1, TLR3, TLR7, IRF3, IRF7, and MAVS genes was induced in Lf-treated Caco-2 cells. The virus infection and replication were also inhibited, partially. It was concluded that Lf showed great potential as a an immunomodulator against the infection of SARS-CoV-2 (60). In another *in-vitro* study from Ward et al. (2022), the human lung cell line H1437 was used to assess the anti-SARS-CoV-2 effectiveness of commercially available bovine Lf and common dairy components. It was shown that bovine Lf demonstrates a broad spectrum of antiviral efficacy *in-vitro* against SARS-CoV-2 variants, including the South African B.1.351, UK B.1.1.7, Brazilian P.1, and Indian Delta variants (61).

The role of Lf as an iron-binding protein has also been studied. The impact of iron on COVID-19 inflammation was discussed by Dalamaga et al. (2020), as well as the possible benefit of iron chelators in lowering SARS-CoV-2 inflammation brought on by iron overload (62).

When considered collectively, all these characteristics allow us to view Lf as a potentially useful tool capable of addressing multiple facets of the viral development and pathogenesis of COVID-19.

### 2.3 Immunoglobulins (Igs)

Igs, as the foremost protein part of BC, acting essential roles in transferring and supporting passive immunity in the newborn calves (63). The biological and immunological effect of BC Igs on human health has been extensively researched and is still being studied (64). Based on the properties such as size and charge of the molecule, amino acid, and also carbohydrate content Igs are classified as IgG, IgA, IgE, IgD, and IgM (19, 65, 66). The most abundant antibody present in BC is IgG and the IgG1 and IgG2 are the two subclasses of IgG which account for 80-85% of the total immunoglobulin content of BC (19, 65–67). In comparison to mature milk, the Igs, the main group of immunological components found in BC, are approximately 100-fold greater (68). Based on the reports from several researchers, the higher concentrations of IgG present in BC demonstrate a variety of immuno-protective and immuno-modulatory features (19, 65, 66, 69).

Generally, Igs are widely recognized molecules for immunogenic response-related health benefits in human diseases. The basic mechanism of the immunogenic response of Igs is when foreign molecules (antigens) enter the host organisms, the Igs bind and eliminate these antigens such as bacteria, viruses, toxins, etc. When the host is exposed to the same antigens, they promote the formation of antibodies to eradicate the illness from the body (70, 71). There are a lot of research using this antiviral mechanism in the literature (72).

In the 1970s, Ellens et al. made the initial discovery that normal cow’s milk contains bovine IgG1 antibodies against rotavirus (73). Then, a number of studies on the binding of bovine IgG to numerous human bacterial pathogens and viruses, such as Helicobacter *Campylobacter jejuni (C. jejuni)*, *Cryptosporidium*, *Klebsiella pneumoniae*, *Salmonella typhimurium*, *Streptococcus*, and Rotavirus (74–76). Bovine Igs are also effective against respiratory pathogens like the human Respiratory Syncytial Virus (RSV), the influenza virus, and *Streptococcus pneumoniae* in addition to detecting other types of pathogens (77).

Numerous alternative approaches have been employed to combat the viruses and also SARS-CoV-2. Hyperimmune BC (HBC), produced by immunizing cows during gestation, contains a high amount of targeted Igs that can be used for the treatment of several infections. The usage of HBC against viruses is well-documented in the literature. Purcell et. al., 2012 developed the vaccination and production system to produce large-scale neutralizing HIV-1 IgG from recombinant HIV-1 gp140 oligomers vaccinated cows. It was resulted that HBC and also IgG derived from BC exhibited neutralizing properties against the variety of Env-pseudo typed viruses (78). NG et. al., 2010 showed the hemagglutination-inhibitory activity of IgG and F(ab’)2 derived from the HBC of influenza-vaccinated cows (79).

Therefore, the same strategy was employed against COVID-19 infection taking into account prior research on the preventative and therapeutic effects of BC-derived Igs on other pathogenic pathogens (79–83). In COVID-19 patients, the immune system is so important, and BC which has a considerable amount of Igs is crucial for boosting the immune system (84). The published studies regarding the usage of HBC suggest that it is a potential adjuvant for COVID-19 patients because of its strong safety profile (85).

HBC, which has been shown to be effective against different viral infections, was produced for immunization with inactivated SARS-CoV-2 and tested in clinical trials. After measurement of the IgG level found in sera against the virus and the level of neutralizing antibodies in the colostrum and sera using ELISA tests, the product’s safety was assessed in 40 healthy volunteers between the ages of 18 and 65. The BC samples had a titer of the neutralizing antibody that was 69 times higher as compared to sera samples. According to the phase I clinical trial results, there wasn’t any adverse effect or complications in participants and phase II studies are ongoing. HBC was preferred as a treatment strategy of COVID-19 for Nili et. al., 2021 (85).

Considering the activity of BC-derived neutralizing antibodies (NAbs) against the SARS-CoV-2 virus, NAbs have a mechanism that impedes the interaction between the ACE2 and S protein SARS-CoV-2 by blocking the cell entry from viruses. By using this great mechanism, Kangro et. al., 2022 produced NAbs from immunized pregnant cows for the effective blocking of SARS-CoV-2 infection. They also demonstrate the BC-antibody-containing nasal spray for COVID-19 patients. To do so, they concluded the antibodies against the SARS-CoV-2 viruses produced from the BC exhibit significant promise as a preventative medicine and BC Igs have a highly effective neutralizing effect in the *in-vitro* assay (86). To assess HBC’s capacity to stimulate antiviral interferonγ (IFNγ) T cell responses, Ilan et. al., 2021 performed preclinical and clinical studies. In this research, orally administered HBC during the 5 five days resulted in augmentation of the number of antiviral T cells responses to antigens in healthy participants. Thus, it was suggested that the application of HBC shows great potential for the prevention of COVID-19 (87).

### 2.4 Lactoperoxidase (LPO)

LPO is one of the peroxidase enzymes and it is considered an important antibacterial enzyme that is present in BC. The concentration of LPO in BC is 0.02 g/L, whereas in mature milk, it is 0.03 g/L. Based on the researchers’ reports, the LPO level in BC reaches the highest level after 3-5 days post-partum, compared with the initial concentration in BC (88). Thus, BC has higher catalyzed activity as compared to mature milk. LPO as a crucial antibacterial enzyme catalyzes thiocyanate oxidation and generates intermediate molecules that have antimicrobial activity (89).

Considering its LPO activity, it generates oxidation products that are toxic for some types of bacterial species including *Streptococcus mutans, Salmonella typhimurium, Staphylococcus aureus (S. aureus), Pseudomonas aeruginosa*, and *Listeria monocytogenes.* Furthermore, LPO also has a great mechanism that inactivates certain viral species such as HIV, poliovirus, vaccinia virus, etc. (9, 90–92). Of interest, *in-vitro* research on cell models showed hypothiocyanite (OSCN^−^) to be effective at preventing SARS-CoV-2 infection at a micromolar level (93). In another study, Shin et al. (2005) discovered that giving Lf and LPO from bovine milk to BALB/c mice infected with the influenza virus strain reduced inflammatory cell infiltration, suppressed pneumonia, and significantly lowered lung consolidation scores. Furthermore, on Day 6, BLPO dramatically reduced the amount of serum proinflammatory cytokine (IL-6) in mice compared to controls (94). A similar study found that people who did not regularly gargle or wear a face mask but consumed Lf and LPO orally experienced cold and fever symptoms less frequently and for a shorter duration (95). All of these research findings clearly indicate that LPO may also be effective in the treatment of COVID-19.

### 2.5 β-Lactoglobulin (β-Lg) and α-Lactalbumin (α-La)

β-Lactoglobulin (β-Lg) is one of the whey proteins and it makes up around 50% of these proteins (10). By preventing bacterial adherence to the host surface and hindering pathogen colonization, this protein shows antimicrobial properties (96, 97). Its antimicrobial activity is exhibited against several gram-positive and negative bacteria such as *Bacillus subtilis (B. subtilis), S. aureus, E. coli, Bordetella bronchiseptica* (97). Furthermore, β-Lg that has been chemically treated with 3-hydroxyphthalic anhydride may prevent *Chlamydia trachomatis* infection, and both HSV-1 and HSV-2 are susceptible to the 3-HP-lactoglobulin (98).

Peptides derived from α-La serve various biological and physiological functions such as antimicrobial, antiviral, antioxidative, and antihypertensive roles, as well as modulation of the immune system (99, 100). For example, peptides from α-La are mostly active against different microorganisms such as *Staphylococcus epidermidis* ATCC 12228*, Staphylococcus lentus, and B. subtilis BGA* (101).

Overall, these features of β-Lg and α-La suggest that it can be considered as the potential bioactive compound in BC for the treatment of COVID-19.

### 2.6 Lysozyme

A crucial component of the innate immune system, lysozyme has potent antibacterial properties against bacterial, fungal, and viral infections. It boosts the effectiveness of other antibiotics, guards against infections, functions as a natural antibiotic, and fortifies the immune system (102). The concentration of lysozyme differs between the species and BC has 0.14-0.7 mg/L of lysozyme, while mature milk contains 0.07-0.6 mg/L of lysozyme. Age, health, the animals’ parity, and the lactation period are a few of the variables that affect lysozyme concentration in BC (10). There has been a lot of research on lysozymes and their antimicrobial mechanism in literature. By catalyzing the breakdown of the β-1,4 bonds in peptidoglycan in bacterial cell walls, lysozyme exhibits its antibacterial effect (103). Peptidoglycan defines cellular form and provides effective defense against cellular turgor pressure, whereas lysozyme activity results in the loss of its integrity, bacterial cell lysis, and ultimately cell death (104). In addition, rather of acting primarily through its enzymatic function, lysozyme can prevent the growth of viruses and fungus like *Aspergillus niger* and *Penicillium* that are connected to its charge (105).

Regarding the antiviral activity of lysozyme, this bioactive enzyme has been shown to be effective against herpes simplex and herpes zoster (106) and to prevent replication of HIV-1 viruses (107), by assembling compounds containing viral DNA. There are notable findings that treatment with lysozyme aerosol is helpful in lowering inflammation and lung tissue damage in animal models of pneumonia and emphysema in addition to its antiviral effect (108). Additionally, lysozyme has neuroprotective properties that may aid to counteract the neurological effects of COVID-19 (109). In the present coronavirus pandemic, a modified form of lysozyme can be utilized to boost the production of interferon, a powerful anti-coronavirus chemical, and so reduce the danger of the life-threatening type of COVID-19 by up to 79% (110, 111). So, it can be a useful component to develop new strategies for the treatment of COVID-19 (112).

### 2.7 Growth factors

Growth factors are considered signaling proteins that are secreted by various cell and tissue types including neighboring cells, glands, also cancer cells. They can promote cell growth, differentiation, survival, and inflammation, and they are typically regarded as a subgroup of cytokines. For proliferation and viability, growth factors are required for all cell types (113, 114). These bioactive components were first documented in 1997 by Pakkanen and Aalto, and then by Gauther et al. in 2006 (9, 115). Betacellulin GF (BTC), Epidermal GF (EGF), FGF-1, Fibroblast GF (FGF-1, and 2), Insulin-like GF (IGF-1 and 2), Transforming GF (TGF-β1, TGF-β2), and Platelet-derived GF (PDGF) are reported growth factor types that are present in milk and colostrum. EGF, IGF, and TGF growth factors are the most prominent types among the others ones (9).

The transforming growth factor-β (TGF-β) is a type of pleiotropic cytokine that is produced by various cell types such as immune cells and non-hematopoietic cells etc. In the cellular process, it participates in regulating immune responses such as inducing or suppressing immune responses, cell proliferation, and oncogenesis (116–118). TGF-β contributes to the induction of immunological tolerance by suppressing inflammatory responses to luminal bacterial antigens in the intestine (116). Another important activity of TGF-β is the modulation of airway inflammation and effector T helper 2 cell-induced hyper-reactivity. This protein suppresses the airway inflammation that performs a critical regulatory role in asthma (119).

Accumulating evidence demonstrates that this immunomodulatory protein supports the immune development of children and protects them against different types of allergies and possible inflammation (120). Because of such properties, they may be a possible strategy for the prevention of COVID-19 and other types of diseases.

### 2.8 Oligosaccharides (OS)

OS isolated from bovine milk and colostrum are other types of bioactive components that are gaining commercial interest due to their potential health benefits (121). Highly selective and complex OS are found in BC, and up to date, approximately 50 distinctive OS have been analyzed in this content. Emerging studies on OS have revealed significant differences in the composition profile and relative abundance between cows, and these bioactive components were found to be present in BC at a concentration of 0.7-1.2 mg/mL. Therefore, BC is considered a preferred source of OS as compared to mature milk because of its elevated level of OS and also ease of isolation and identification (122–126). Most of these structures are acidic oligosaccharides that are present in mature bovine milk at lower levels (127).

Bioactive OS from bovine milk and colostrum are similar to human milk OS due to their chemical structures and they have great importance because of their significant roles in biological and physiological mechanisms such as prebiotic activity, protection against various pathogens, etc. (127–133). Their main function appears to be serving as competitive inhibitors for the binding sites on the intestine epithelial surfaces to protect against infections (134). Additionally, there is evidence to support the idea that some of these bioactive components act as colonic microflora genera growth promoters. According to Karav et al. (2016), BMO stimulates *Bifidobacterium longum subspecies infantis (B. infantis)* development in the infant’s intestine similar to HMO. Complex and hybrid-type glycans from bovine milk and colostrum show prebiotic activity, similar to HMOs/BMOs, selectively utilized by beneficial microorganisms. Promoting brain development, and alleviating metabolic issues are other health benefits of BMO (135).

Considering the biological activity of oligosaccharides, they found in milk and colostrum can compete with pathogenic bacteria and viruses for attachment sites because they are soluble receptor mimics of the carbohydrates found on the surface of epithelial cells. For instance, HMOs were shown by Andersson et al. (1986) to prevent pneumococci or influenza virus from adhering to pharyngeal or buccal epithelial cells (136). Similarly to this, it has been demonstrated that sialylated oligosaccharides prevent pathogenic *E. coli* strains from adhering to infants (137). In similar studies, OS has been demonstrated to function as receptor analogs for cell surface locations in the digestive tract epithelium, inhibiting the adherence of viruses (138, 139) and bacteria like *Helicobacter pylori* (140).

In addition, researchers have considered a possible source of anti-infective glycans in BC, which is high in neutral and acidic oligosaccharides. Potential anti-infective glycans in naturally obtained foods were looked into. Anti-infective effectiveness against a highly invasive strain of *C. jejuni* was investigated for oligosaccharides extracted and purified from the colostrum of Holstein Friesian cows. Because mucins found in BC are highly glycosylated molecules, this discovery would imply that glycan-based substances may function as anti-infectives for *C. jejuni*. In this context, 37 BC oligosaccharides (BCO) have been structurally characterized by Hydrophilic Interaction Liquid Chromatography-High-Performance Liquid Chromatography (HILIC-HPLC) in conjunction with exoglycosidase digestions and offline mass spectrometry, and it has been shown that *C. jejuni* can bind to some of these constructions *in-vitro*. They period-treated the BCO concurrently with the inhibitory test, suggesting a direct bacterial-oligosaccharide interaction that reduced the anti-infective effectiveness of the glycans. This was proven when the BCO entirely eliminated *C. jejuni’s* ability to bind to chicken intestinal mucus *in-vitro*. This study provides strong evidence for the anti-infective property of oligosaccharides obtained from BC (141).

Thus, OS may have several uses in the culinary and pharmaceutical industries due to their bioactive properties. In order to comprehend the connection between OS structure and biological function, it is crucial to analyze their structure (128). These qualities make them potentially useful approaches for the SARS-CoV-2 prevention.

### 2.9 Glycosaminoglycans

Glycosaminoglycans (GAGs), commonly referred to as mucopolysaccharides, are negatively charged, sulfated linear polysaccharide molecules found in milk. Examples include heparin/heparan sulfate (HP/HS), hyaluronic acid (HA), chondroitin sulfate (CS), and dermatan sulfate (DS). In the mammary glands, a particular core protein is connected to the lengthy chains of milk GAGs during production (142).

Milk GAGs may play two crucial roles in the context of COVID-19. These GAGs are used as prebiotics by microorganisms in the intestinal system to promote growth (143). This helps preserve gut health by preventing various enteric bacterial and viral illnesses. Second, although some viruses have been shown to be resistant to the antiviral effects of commercially available GAGs, such as the dengue virus (144), HSV (145), Zika virus (146), and SARS-CoV (147), commercial CS has not been proven effective against all viruses. It is essential to fully describe the antiviral potential of GAGs in bovine milk. Additionally, the fact that several GAGs share similar carbohydrate structures may account for their viral inhibitory effect against various viruses. According to reports by Kwon et al. (2020), free HS prevents Vero cells from becoming infected with SARS-CoV-2. GAGs may have antiviral activity against SARS-CoV-2, the infectious agent responsible for the COVID-19 pandemic (148). Future research may be interesting to further examine and assess the potential of BC and milk GAGs to inhibit this deadly virus.

### 2.10 Milk fat globule membrane (MFGM)

Mammary epithelial cells release milk fat globules, which are encased in a complex membrane that is called the milk fat globule membrane (MFGM). Polar lipids such as phospholipids and sphingolipids, membrane proteins including glycoproteins, and also enzymes are some of the several substances that make up the thin trilayer structure of MFGM (149–151). The isolation, processing, and analysis methods used as well as the breed of the cow affect the protein and lipid contents of MFGM (12).

Recent research has suggested that MFGM proteins have nutritional and practical benefits for people such as supporting the composition of healthy gut microbiota, protection against infection and inflammation, etc. (152–154). Considering the MFGM proteomes of BC and its importance for the well-being of neonatal calves, it is a rich source of bioactive proteins. Further characterization of these bioactive proteomes can provide important insights into the health benefits of human well-being. Given that several proteins in the bovine MFGM have been demonstrated to have inhibitory effects on a variety of pathogens consuming whey protein concentrate enriched with MFGM may help prevent diarrhea that is both bacterial and viral in origin (155).

Information on the presence of MFGM derived from BC and milk demonstrated that mucoprotein 1 (MUC1), butyrophilin, and lactadherin are a potential bioactive fraction of MFGM. For instance, it was hypothesized that lactadherin might be responsible for the function of the mucin complex based on research with virus-binding properties (156). Another research team carried out *in-vitro* tests to look for rotavirus-inhibitory elements in bovine milk. One component of bovine whey protein with a high molecular weight showed antiviral activity. Lactadherin, MUC1, and an unknown 80-kDa protein were all present in this fraction. Xanthine oxidase (XO), one of the main MFGM enzyme proteins, has been demonstrated to have antibacterial and immunoprotective effects (157, 158). Butyrophilin, the most prevalent MFGM protein, is known to have immune-modulating properties such as anti-infective or anti-inflammatory actions (159, 160).

Morever, the biological roles of lipid fractions in MFGM have been extensively studied. This bioactivity includes preventing pathogenic and viral infections, reducing cholesterol-induced steatosis, and maintaining gut health (161). It has been shown that MFGM isolates boost resistance to rotaviral infection. In a study looking at MFGM produced from both buttermilk and whey cream, rotaviral infectivity was assessed using a fluorescence focus test. MFGM was shown to exhibit anti-rotaviral effects in all fractions in a dose-dependent manner, while the isolate from cream was marginally more potent (162). This component most likely has a more varied lipid content, which might work in concert with the related viral proteins. In an *in-vitro* investigation where bovine and ovine MFGM sources were investigated, it was further shown the importance of the lipid fraction in MFGM for preventing rotaviral infection. The cream-derived components in this investigation were discovered to have a rotaviral-neutralizing effect. Nevertheless, heat treatment (denatured proteins) and cream washing (reduced lipid content) both result in reduced effectiveness (163).

As a result of the findings of the possible functional and physiological properties of this component, MFGM can be exploited against viruses in the pharmaceutical industry.

### 2.11 Vitamin and minerals

Vitamins and minerals are two essential components crucial for a healthy metabolism, growth, and development. They serve as coenzymes throughout the body, and because they are naturally balanced and delivered in colostrum according to the body’s needs, they play a vital role in maintaining health. Specifically, BC contains both fat-soluble vitamins (A, D, and E) and water-soluble vitamins (B series and C), which could have significance for various metabolic functions, such as supporting bone development and acting as antioxidants. Additionally, vitamin D has been linked to improved immune system and mental wellness (14). Compared to mature milk, BC often has higher concentrations of most vitamins, including vitamins B2, B12, E, and D.

In accordance with a different scientific study, vitamin A found in cow’s milk may affect lymphocytes’ homing to the upper respiratory system by inducing the production of tissue homing-linked markers α4β7 (164). Additionally, vitamin A has been suggested as a substitute for CoV therapy and as a strategy to avoid lung infections. Jee et al. (2013) found that calves who received insufficient vitamin A had a reduced ability to produce antibodies in response to the inactivated BCoV vaccine (165). Therefore, vitamin A may play a supporting role in the management of COVID-19, in conjunction with the development of a favorable antibody response in those who have received the CoV vaccination. Numerous studies have shown that vitamin D can lower the incidence of pneumonia and acute viral respiratory tract infections (166, 167). Possible mechanisms of action include the prevention of viral replication, anti-inflammatory effects, and immunomodulatory effects (168). Studies have demonstrated that vitamin D decreases the overexpression of proinflammatory cytokines (TNF-α, IL-1, IL-1β, and IFN-γ), increases the expression of anti-inflammatory cytokines, and releases defensins and cathelicidins that halt viral replication (169). These findings suggest that vitamin D may expedite the recovery of patients with viral infections, particularly COVID-19 (170). The antibacterial and antioxidant properties of vitamin D, along with its role in enhancing the immune system’s resistance to lung infections and airway inflammation, make it a potentially effective treatment for SARS-CoV-2. Free radicals, reactive oxygen species (ROS), and reactive nitrogen species (RNS) are neutralized by the fat-soluble antioxidant vitamin E through the donation of hydrogen ions from its chromanol ring (171). As a result, it reduces oxidative stress, which is primarily responsible for acute respiratory distress syndrome, especially in the case of COVID-19 (172). One of the main causes of death in COVID-19 patients is ferroptosis, which is caused by a deficiency in vitamin E and results in various injuries to the heart, liver, kidneys, intestines, the neurological system, and other organs (173). Vitamin K plays a significant role in the pathogenesis of COVID-19, and its deficiency is linked to numerous organ damages, thrombotic complications, and high mortality (174). Vitamin K insufficiency during the initial stages of COVID-19 infection has been associated with increased IL-6 production and Th2 storm activation, according to research (175).

According to research, vitamin B12 has a high affinity for the SARS-CoV-2 protease (176). Additionally, a deficiency in vitamin B12 results in symptoms comparable to COVID-19, such as increased oxidative stress, elevated homocysteine levels, thrombocytopenia, elevated lactate dehydrogenase, low reticulocyte counts, vasoconstriction, and renal and pulmonary vasculopathies (177). Thus, symptoms of COVID-19 may be alleviated by vitamin B12. Vitamin C has antiviral properties, including reducing endothelial dysfunction, increasing and controlling the generation of IFN and cytokines, reducing inflammation, and restoring mitochondrial function (178).

Besides being rich in a number of vital elements, such as calcium, copper, iron, zinc, magnesium, manganese, and phosphorus, BC is also rich in mature milk (34, 68). Their concentration, however, varies widely and is influenced by numerous factors (179). Minerals might play a crucial role in the treatment and prevention of COVID-19. For instance, normal respiratory function, energy production, immune system strength, nerve conduction, blood coagulation, control of heart rate, hormone and enzyme secretion, and muscular contraction all depend on calcium (180). Numerous researchers have noted that individuals with COVID-19 frequently exhibit a high frequency of hypocalcemia (181). The worst clinical outcomes, including multiple organ dysfunction syndrome, septic shock, and mortality, were observed in COVID-19 patients with low serum calcium levels, particularly below 2.0 mmol/L (182). In addition to regulating fluid and electrolyte balance, sodium is crucial for maintaining normal cellular homeostasis. Studies on rats have shown that a high-sodium diet leads to a decrease in the expression of ACE2 in renal tissue (183). Additionally, it has been found that the COVID-19 disease progresses more severely when serum sodium concentrations are lower (184). This is accompanied by greater IL-6 production. Therefore, salt has a significant influence on the therapeutic outcomes of COVID-19 patients. It is suggested that sodium consumption levels be monitored in patients with severe COVID-19 infections and that low sodium intake be addressed as soon as possible (185). All species require copper as a micromineral, which is important for immune system functioning, protection against free radicals, and respiration (186). Copper improves cell-mediated immunity, the development of certain antibodies, and the functioning of T helper cells, B cells, neutrophils, NK cells, and macrophages in the fight against pathogenic microbes (187). Additionally, copper controls IL-2 levels, NK cell cytotoxicity, the balance between Th1 and Th2 cells, and T helper cell proliferation, all of which are crucial for addressing immunological dysregulation in COVID-19 patients (188). Copper can also downregulate the expression of NF-κB, which is typically stimulated by virus-induced ROS, to reduce the production of inflammatory cytokines, chemokines, and adhesion molecules (189). This could be particularly helpful during the COVID-19 cytokine storm syndrome phase.

In conclusion, in addition to the primary therapy during COVID-19, a number of vitamins and minerals may have symptomatic and supporting functions. Therefore, future research can focus on characterizing these components in BC and exploring their combined use with other bioactive compounds to enhance protection against infections and immune system modulation (190, 191).

## 3 Bovine colostrum applications in virus infections

### 3.1 Bovine colostrum for protection against respiratory infection

Upper respiratory tract infections (URTIs) are common types of tract infections caused by respiratory viruses such as SARS-CoV-2 that affect the respiratory system organs including mouth, nose, throat, larynx, and also trachea and can cause nasopharyngitis, sinusitis, pharyngitis, laryngitis and laryngotracheitis (192). The known symptoms of URTIs are considered sore throat, fatigue, headache, runny nose, and watery eyes (193).

Such findings of studies show that BC can decrease the incidence of URTI and symptoms of infection. In a clinical trial study by Brinkworth and Buckley, 2003, the effects of concentrated BC (CBC) protein on the incidence and self-reported symptoms duration of URTI were assessed. After an experimental process, the results of 60g/day CBC supplementation and 60 g/day whey protein supplementation were compared and they demonstrated there weren’t any differences in self-reported symptoms duration, statistically, whereas CBC supplementation reduced the URTI incidence, significantly (194). Patel and Rana, 2006, assessed how effective and tolerable BC is for the prevention of recurrent URTIs and diarrhea in children. According to their findings, 3.0 g of BC supplementation was found to be effective to reduce episodes of URTI and diarrhea (91.19% and 86.60% of patients, respectively). Supplementation was also found to be an option for the improvement of well-being (195). A similar study from Saad et al. resulted that the intake of BC was enabled to reduce URTI and episodes of diarrhea in 1-6 aged children who received BC supplementation (3.0 g to 6.0 g based on the aged) during 4 weeks (196).

To investigate the benefits of BC for the treatment of URT in children with IgA deficiency, patients received BC or placebo randomly three times each day during the one week. During this clinical study, salivary IgA level of children was detected before/after administration, between the doses of BC because salivary IgA and resistance to URT are directly correlated. It was reported that lower severity of the infection score was observed in the BC group than in the placebo group within a week (0.81 ± 0.83, 3.00 ± 1.85; P = 0.000, respectively). On the other hand, no differences between the groups’ salivary IgA levels were observed. Thus, researchers concluded that to validate the effectiveness of BC taken in patients who have IgA deficiency, more research and clinical study are required (197). Alfayoumi et al., 2020 conducted research along with numerous clinical investigations to examine the safety and effectiveness of the BC regimen in preventing URTI recurrence as well as the influence of BC on the nasal flora. In this case report, there were no URTI episodes over the year after starting the BC regimen as opposed to 5 incidents over the year before the BC regimen (*p < 0.05*). The findings of this case study show that BC was successful in preventing URTIs (198). These outcomes line up with those seen in past research (194, 199).

It is clear from looking at the clinical trials that have been done in this field that researchers have obtained promising results. A randomized, triple blind, placebo-controlled trial study was designed to determine whether the intervention could also help to other young adults who might have been at a higher risk of getting URTIs. For the duration of the study, medical (MED) and health science (HSci) students who were deemed to be at risk received a relatively small daily dose of either BC or a placebo for 45 days. On day 87, a second supplementation phase began and lasted for 7 days. The subjects were only observed for the course of the 107-day experiment by daily online questionnaires. These surveys asked questions regarding the subjects’ overall health, the frequency and severity of URTIs, and any probable gastrointestinal side effects. A significant reduction in the frequency of symptomatic days was seen in the group supplemented with BC compared to the placebo group, particularly among MED students compared to health HSci students, indicating a significant level of protection against URTIs. The intensity of symptoms as well as the general sense of well-being also showed a comparable effect. According to the trial results, BC supplementation can improve defense against URTIs, especially for people at high infectious risk (200).

The information gathered here suggests that BC can function as an adjuvant in preventing lower respiratory tract infections in all ages group. To assess the therapeutic potential of BC supplementation, research on the effects of colostrum supplementation on respiratory tract infections is required. Altogether, BC has gained importance with research based on Exercise Nutrition and Immunology.

### 3.2 Evidence for protective effects of bovine colostrum in enteric virus infection and SARS-CoV-2

BC has gained importance because of its important features, and it can be employed as a novel approach for the treatment of a variety of diseases in human beings. It is also considered a supportive component in terms of maintenance of different systems such as the gastrointestinal system, local/systemic immunity, etc. Additionally, nonsteroidal anti-inflammatory drugs (NSAIDs)-induced intestinal damage has been treated and prevented with the use of BC. It has been successfully used to treat a variety of joint ailments, including rheumatoid arthritis, and shows immune modulator properties for the treatment of allergic and auto-immune diseases. The use of BC in preventing and treating bacterial and viral infections is supported by several clinical observations (56, 57).

A key feature of BC is that BC and its bioactive components are beneficial to take care of potential upper respiratory tract infections, sinusitis, and pneumonia. In physiological and biological processes, BC is a promising approach for the prevention and treatment of COVID-19 because of its antibacterial/antiviral activity and its capacity to boost both the innate and adaptive immune systems of humans (191).

Systematic reviews, *in-vitro* and clinical studies reported that supplementation of BC against COVID-19 can reduce the rate of incidence, and disease symptoms and prevent infection of SARS-CoV-2. For the prevention of SARS-CoV-2 infection using a nasal spray strategy, researchers conducted a clinical study. In this trial, the effectiveness of a virus-neutralizing BC supplement delivered intravenously in preventing SARS-CoV-2 infection in household contacts of SARS-CoV-2-positive people was assessed and this trial is still recruiting (NCT number: NCT05552950).

The effect of BC was investigated for evaluation of early recovery in COVID-19 patients by Khartode, 2021. Khartode, 2021 categorized the patients based on their symptoms including mild, moderate, and severe using HRCT Chest Scan results and symptoms’ severity. According to statistical analysis, the early recovery for all symptom categories in the study group was found as significant (p<0.05). Khartode, 2021 resulted that BC intake could be beneficial for COVID-19 patients and their early recovery (84).

A double-blind, randomized control study was conducted to examine the effectiveness of a hen egg white and BC supplement in preventing severe COVID-19. Adults who had mild to moderate COVID-19, risk factors for severe disease, and had experienced symptoms during the last 5 days were split into two groups for this study: the intervention group (n = 77) and the placebo group (n = 79), who randomly assigned. Up to 42 days after enrolment, symptoms were monitored, and viral clearance was assessed 11-13 days after the onset of symptoms. Based on the study records, one participant out of the total number of subjects experienced severe COVID-19. The active arm displayed a decreased severe-type symptom score at 11 to 13 days following the beginning of symptoms in comparison to the placebo group (p = 0.049). In conclusion, the results show that when hen egg white and BC were combined, symptoms in those with mild or moderate COVID-19 were less severe. (Clinical Trial Registration: DOH-27-062021-9191) (201) **Table 2**.

**Table 2:** Bovine colostrum and its bioactive components against viruses.

Bovine Colostrum and Its Bioactive Components	Study Type	Name of the Virus	Mechanism of Action	References
**Immunoglobulins**	*In-vitro and In-vivo*	HIV	Cross-clade binding and neutralization activity against virus	(78)
	*In-vivo*	Influenza	Neutralization activity against virus, Increasing NK cell activity and blocking would reduce interleukin 6 production by epithelial cell	(79, 81)
	*In-vivo*	RSV	Inhibition and amelioration the symptom by RSV infection, and enhance the CD8 T cell responses	(80)
	Clinical Study	SARS-CoV-2 NCT04643561	Activation of a systemic antiviral immunity by targeting the gut immune system.	(87)
**Oligosaccharides**	*In-vitro*	*Campylobacter jejuni* Infections	Reduction of the invasion and translocation of enteric infection/virulence effect	(141)
**BC**	Clinical trial	Respiratory Tract Infections	Evaluation of the effects of a BC on the early recovery of COVID-19 patients across mild, moderate, and severe categories	(84, 194–198, 200)
	Clinical trial	HIV	Increasing nutritional support and immunity	(83, 202)
	Clinical trial	SARS-CoV-2	Neutralization activity against virus Enhancement of resistance to the development of symptoms	(85, 86, 201)

**Figure 2:**
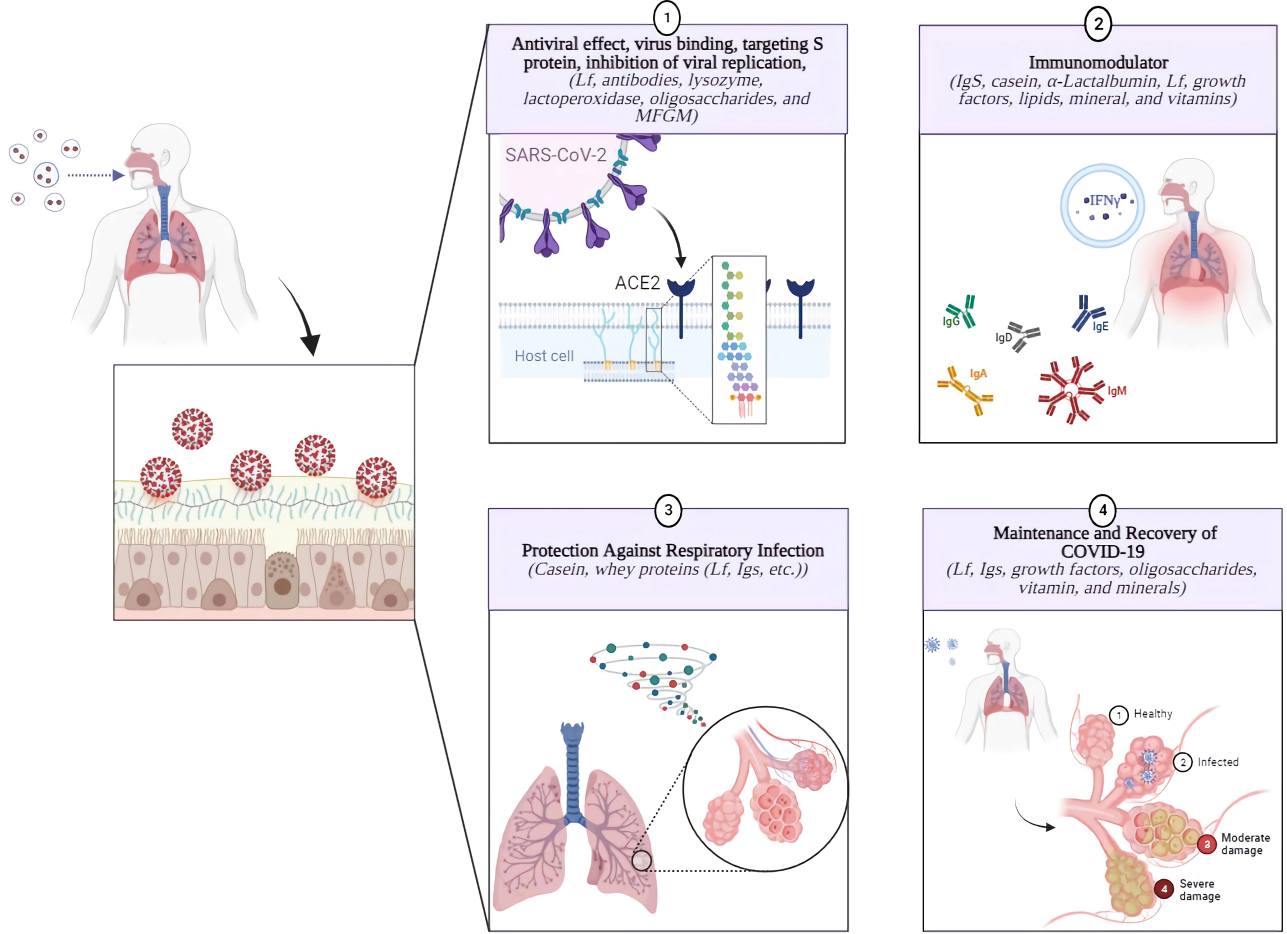
Bioactive Components of Bovine Colostrum and Their Significant Effects on Prevention and Management of COVID-19. BC may be considered a viable method to assisting in the treatment of SARS-CoV-2 patients or protection from the infection. BC has immunomodulatory effects due to its bioactive components, such as Igs, Lf, caseins, growth factors, among others, which may help modulate immune responses. Furthermore, its bioactive components, including Lf, antibodies, lysozyme, LPO, oligosaccharides, MFGM, etc., have a potential antiviral activity against SARS-CoV-2 and respiratory infections. Its comprehensive food profile is also effective in post-illness recovery by supporting a healthy immune system by providing proteins, oligosaccharides, vitamins, minerals, and amino acids (3, 12, 203).

Thus, a variety of *in-vivo*, *in-vitro*, and clinical trial data support the effectiveness of BC for the prevention of COVID-19 as an immune-boosting supplement (**Figure 2**). Additional efforts are needed to explore new uses and approaches in developing innovative BC-based treatments for COVID-19.

## 4 Concluding remarks and outlook

The multifunctional effects of BC, including its antiviral, anti-inflammatory, and immunomodulatory properties, have generated a great deal of research over the years. Particularly in relation to viral diseases such as COVID-19, these qualities have garnered a lot of interest. The results of investigations on the emergence and management of COVID-19 have shown that BC and its bioactive components have promise as both non-pharmacological and pharmaceutical treatments for COVID-19.

Inhibiting virus infections and reducing the intensity of illness symptoms are two of BC supplementation’s main benefits. Because of its high concentration of bioactive components such as Igs, several immune boosting factors, BC has immunomodulatory effects, potentially supporting a balanced immune response. Additionally, its bioactive components may cross-react with SARS-CoV-2, assisting in virus neutralization, and its comprehensive dietary profile provides essential vitamins, minerals, and amino acids, promoting a healthy immune system. This shows that BC might be an effective preventative measure against SARS-CoV-2 infection, lowering the chance of infection or lessening its effects if infection does happen. It is crucial to stress that any usage of BC for COVID-19 prevention or management should be done so under the guidance of a qualified medical expert to ensure safety and efficacy.

There is a need for additional research into the creation of BC-based products given the significant medicinal potential of BC and its bioactive components. Infections caused by other viruses as well as COVID-19 may be successfully treated with these products. Investigating the potential of BC becomes even more important in the context of public health emergencies, like as pandemics, where quick and efficient preventive actions are essential.

The use of BC and its bioactive ingredients is not just restricted to the medical field. Products with BC-derived ingredients can be beneficial for a variety of sectors, including the pharmaceutical and food sectors. The adaptability of BC makes it a desirable choice for creating novel products with potential health advantages. Additional scientific study is crucial to advancing the use of BC and its bioactive components. To prove its safety and effectiveness in the prevention and treatment of COVID-19 and other disorders, it is required to investigate its mechanisms of action, optimize dosages, and carry out clinical trials.

In conclusion, BC is a promising choice for further scientific investigation due to its multifunctional qualities and potential as a different strategy to combat COVID-19. Other industries might gain from its implementation, which could go beyond the medical sector. To assure the efficiency and safety of BC-based products in treating public health emergencies and enhancing overall health outcomes, it is required to approach their creation and use with a rigorous scientific research and expert supervision.

## Author contributions

HD and SK both contributed significantly to the work’s conception, design, literature searches and interpretation, and HD drafted the article and SK provided critical revision of the article; both HD and SK have read and approved the manuscript. All authors provided comments, direction, and advice in updating manuscript drafts to the final version. All authors contributed to the article and approved the submitted version.
